# Minimizing off-Target Mutagenesis Risks Caused by Programmable Nucleases

**DOI:** 10.3390/ijms161024751

**Published:** 2015-10-16

**Authors:** Kentaro Ishida, Peter Gee, Akitsu Hotta

**Affiliations:** 1 Center for iPS Cell Research and Application (CiRA), Kyoto University, 53 Kawahara-cho, Shogoin, Sakyo-ku, Kyoto 606-8507, Japan; E-Mails: kentaro.ishida@cira.kyoto-u.ac.jp (K.I.); peter.gee@cira.kyoto-u.ac.jp (P.G.); 2 Institute for Integrated Cell Material Sciences (iCeMS), Kyoto University, Yoshida Ushinomiya-cho, Sakyo-ku, Kyoto 606-8507, Japan

**Keywords:** CRISPR Cas9, genome editing, mutagenesis, off-target effect

## Abstract

Programmable nucleases, such as zinc finger nucleases (ZFNs), transcription activator like effector nucleases (TALENs), and clustered regularly interspersed short palindromic repeats associated protein-9 (CRISPR-Cas9), hold tremendous potential for applications in the clinical setting to treat genetic diseases or prevent infectious diseases. However, because the accuracy of DNA recognition by these nucleases is not always perfect, off-target mutagenesis may result in undesirable adverse events in treated patients such as cellular toxicity or tumorigenesis. Therefore, designing nucleases and analyzing their activity must be carefully evaluated to minimize off-target mutagenesis. Furthermore, rigorous genomic testing will be important to ensure the integrity of nuclease modified cells. In this review, we provide an overview of available nuclease designing platforms, nuclease engineering approaches to minimize off-target activity, and methods to evaluate both on- and off-target cleavage of CRISPR-Cas9.

## 1. Introduction

Genome engineering technologies can be designed to recognize and induce double stranded breaks (DSBs) at specific DNA sequences, which are mainly repaired by two host DNA repair pathways. The nonhomologous end joining (NHEJ) pathway, which induces insertions or deletions (indels) at the cleavage site, can disrupt gene open reading frames, and the homologous recombination (HR) pathway which, in the presence of a complementary DNA sequence, exchanges damaged DNA with a homologous sequence. These technologies have allowed scientists to investigate the functional roles of genes in cells and live animals via gene knockout or knock-in approaches. In recent years, three types of endonucleases, namely ZFNs (zinc finger nucleases), TALENs (transcription activator like effector nucleases), and the CRISPR (clustered regularly interspersed short palindromic regions) associated 9 (Cas9) system have been predominantly utilized for gene editing. However, due to technical challenges involved in constructing specific ZFNs and TALENs, the CRISPR Cas9 system has emerged in recent years as the nuclease of choice because of its ease of construction, by simply designing RNA with a 20 base pair complementary sequence to a targeted region in the genome.

The CRISPR Cas9 system was first identified as an adaptive immune host defense mechanism in bacteria against invading pathogens such as bacteriophages [1,2,3]. The system functions by incorporating DNA fragments of pathogens from past infections into specialized regions of the bacterial genome that are transcribed into crRNA (CRISPR RNA) and bind in tandem with tracrRNA (transactivating CRISPR RNA) to Cas9 endonucleases in a complex that acts as a sentinel for potential threats with complementary DNA sequences [1]. Successful targeting induces DSBs in the foreign DNA, thereby eliminating the threat.

Since its discovery in 2012, the CRISPR Cas9 system has been harnessed into a powerful genome editing tool for scientists to modify the genomes of cell lines [4,5,6,7] and even living organisms (*C. elegans* [8,9], zebrafish [10], mice [11,12], rat [13,14], rabbits [15] and monkey [16]), with relative ease, compared to TALENs and ZFNs. The CRISPR Cas9 system also has tremendous therapeutic potential [17] and has been shown to correct genetic mutations [18] such as β-thalassemia [19], cystic fibrosis [20], hemophilia A [21], cataracts [22], hereditary tyrosinemia type I [23], and Duchenne muscular dystrophy [24]. Furthermore, it has been implemented for host defense in human cells as a tool to eradicate chronic virus infections including hepatitis B [25,26] and HIV [27,28,29].

However, given the early success of Cas9 thus far, accumulating lines of evidence point to a variable level of nonspecific activity at off-target sequences that has caused concern, particularly in the event that these nucleases are used in the clinical setting because the effects could be permanent and detrimental for cellular function, resulting in possible cellular toxicity or even tumorigenicity. The phenomena of non-specific cleavage by nucleases leading to cellular toxicity is not unique to CRISPR Cas9 as it has been also reported for ZFNs and TALENs [30,31,32,33,34], but the parameters directing RNA mediated sequence recognition and cleavage by Cas9 are unique because the targeting mechanism is determined by a complementary RNA sequence [35]. In this review, we will highlight strategies for minimizing off-target cleavage by using available online tools for targeting design, modified Cas9 enzymes to attenuate off-target activity, and cover methods to assess off-target cleavage activity.

## 2. The Structure of Cas9: How it Recognizes and Cleaves DNA

**Figure 1:**
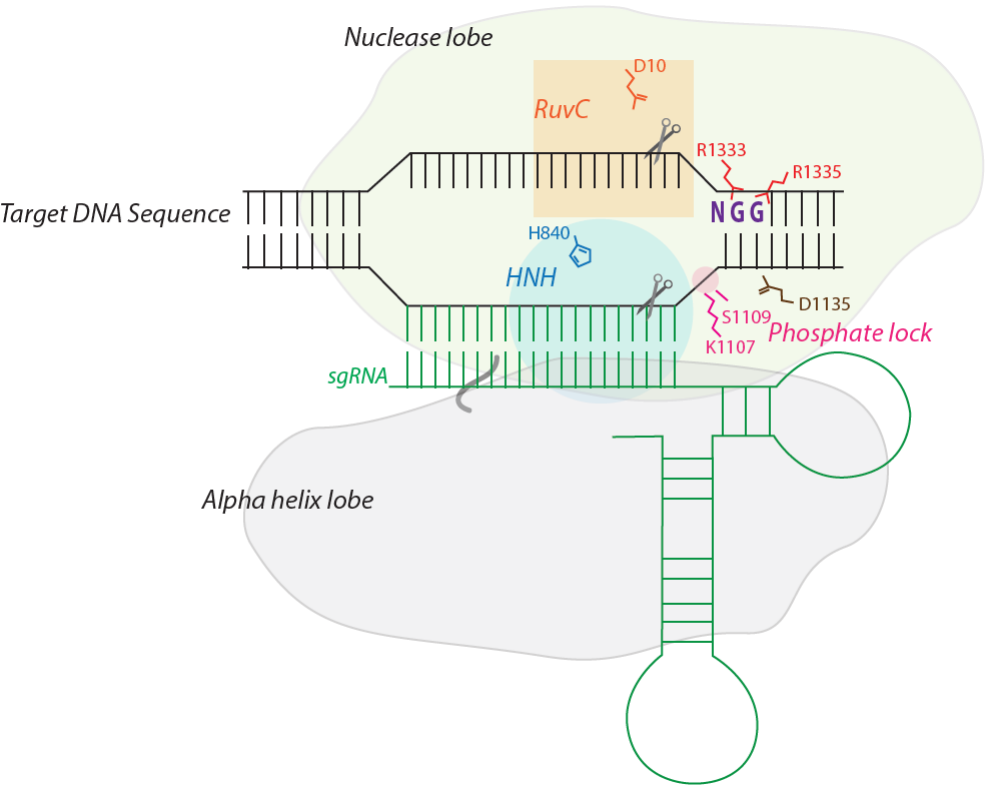
*Streptococcus pyogenes* (Sp) Cas9 recognition of target DNA. Sp Cas9 consists of two lobes, one containing the HNH and RuvC nuclease domains, and an α helix lobe. Arginine residues (R1333 and R1335) of Sp Cas9 recognize the dinucleotide guanine bases of the “NGG” protospacer adjacent motif (PAM). Then, serine (S1109) and lysine (K1107) form a phosphate lock by interacting with a phosphate residue adjacent to the PAM site, which triggers the melting of the double stranded DNA so that sgRNA (green) can form Watson–Crick pairing with a complementary DNA strand. D10 and H840 are the main catalytic residues in the HNH and RuvC nuclease domains, respectively. Aspartic acid at 1135 (D1135) interacts with the third PAM guanine residue through a water molecule, and a modification of the residue to glutamic acid (D1135E) has been shown to improve the recognition of PAM sequence and target specificity.

The crystal structure of Cas9 derived from *Streptococcus pyogenes* (Sp) has been resolved by several groups, and has helped to elucidate the atomic-level interactions between a complex of Cas9, crRNA, trascrRNA and target DNA, and the dynamic conformational changes the protein undergoes during substrate binding and cleavage [36,37,38]. Essentially, a Cas9 protein consists of two regions shaped like large lobes, one of which harbors two nuclease activities, RuvC and HNV, and the other, an α helix (Figure 1). The main catalytic residues responsible for cleavage activity in RUH and HNV are D10 and H840, respectively (Figure 1). Alone, Cas9 exists in an inactive state that undergoes conformational change upon guide RNA (sgRNA) binding, causing its two lobes to pull apart from one another and create a channel that is large enough to accommodate a target double strand DNA and sgRNA (Figure 1) [36]. Amino acids lining the channel are positively charged and interact with the phosphate backbone of the nucleic acids.

If a trinucleotide “NGG” PAM (protospacer adjacent motif) exists adjacent to the 3′ end of the target sequence on the noncomplementary strand, then a PAM interacting domain (R1333 and R1335) binds to the “GG” dinucleotide sequence (Figure 1) [39]. D1335 is another residue that interacts with the third PAM guanine via a water molecule. Opening of the dsDNA from the PAM site exposes a phosphate at the adjacent position, which interacts with lysine and serine residues (K1107 and S1109), termed a phosphate lock (Figure 1) [39]. This interaction triggers the unzipping of the double stranded DNA so that crRNA can form Watson–Crick pairing with the complementary DNA strand [39]. Cleavage of each DNA strand occurs by the RuvC and HNH nuclease domains, respectively.

## 3. Designing sgRNA to Target a Genomic Target Site

For experimental purposes employing the use of Sp Cas9, crRNA and tracrRNA can be combined into a single guide RNA (sgRNA), where crRNA contains a 20 bp complementary sequence to a targeted genomic DNA region and the tracrRNA acts as a scaffold. The 20 bp complementary sequence should be followed by an “NGG” trinucleotide PAM for Sp Cas9 binding and cleavage to occur, but non-canonical PAMs (“NAG”, “NTG”, and “NGA”) are also sometimes recognized [40]. Furthermore, Cas9 may be able to bind to a sequence with as many as 10 mismatches in the spacer of crRNA [41], yet only cleaves and induces mutations at sequences with 3–5 mismatches [42]. Previous reports have termed the PAM and PAM adjacent sequence (3′ half of the spacer) the seed sequence because this DNA region is scanned by the Cas9-sgRNA complex from the PAM side [43]. The seed sequence is critical for cleavage specificity and is less permissive for mismatches, while some mismatches in the non-seed region (5′ half of the sgRNA target sequence) may be tolerated for Cas9 binding and DNA cleavage. Due to this nonspecific nature, selecting a target sequence that has minimal overlap with other sequences located next to a canonical and non-canonical PAM is crucial for the initial sequence recognition and specificity. Therefore, CRISPR sgRNA should be designed to target a highly unique genomic region.

## 4. Tools to Design Highly Specific sgRNA

To design specific sgRNA, it is critical to evaluate the number of potential off-target sequences in the genome. There are now a growing number of web tools available to aid in the identification of targetable sites for CRISPR sgRNAs and evaluate their specificity (Table 1). Each sgRNA design tool has its own unique properties and algorithms, but most first search for PAM sequences in a specified genomic sequence or in a region defined by an accession number according to the target region size. CRISPR Design [6,44], selects sgRNA from an input sequence up to 250 bp, while CRISPRdirect [45,46], ZiFit Targeter [47,48], Cas9 design [49,50], E-CRISP [51,52], and CHOPCHOP [53,54] can perform searches from much longer target regions.

**Table 1:** Software to design CRISPR (clustered regularly interspersed short palindromic regions) sgRNA (single guide RNA).

Tool	Organism	Input (Length)	off-Target Sites	Reference
ZiFiT	Hs, Rn, Mm, Dr, Dm, Ce, Aa, Ec	Target sequence (<1000 bp)	Mismatches	[48]
CRISPR Design	>15 species	Target sequence (<250 bp)	Mismatches	[6]
Cas9 design	>10 species	Target sequence (>10 kbp)	Mismatches	[50]
E-CRISP	>15 species	Target sequence (>10 kbp), gene name	Mismatches	[52]
CasOT	Any species	Target sequence (>10 kbp)	Mismatches	[55]
Cas-OFFinder	>10 species	Designed sgRNA (10–25 nt)	Mismatches, insertion and deletions	[56]
CHOPCHOP	>20 species	Target sequence (>10 kbp)	Mismatches	[54]
GT-Scan	>20 species	Target sequence (<4000 bp)	Mismatches	[57]
sgRNAcas9	Any species	Target sequence (>10 kbp)	Mismatches	[58]
CRISPR-P	>20 plants	Target sequence (<5 kbp)	Mismatches	[59]
COSMID	Hs, Mm, Rn, Ce, Mam, Dr	Designed sgRNA (10–55 nt)	Mismatches, insertions and deletions	[60]
sgRNA Designer	Hs, Mm	Target sequence (<10 kbp), gene ID	N.A.	[61]
iGEATs	Hs, Mm	Chromosomal locus, target sequence (<25 kb), gene name, gRNA	N.A.	[17]
CRISPRdirect	>15 species	Target sequence (<10 kbp)	Mismatches, insertions and deletions	[46]
CRISPR-ERA	Hs, Mm, Rn, Dr, Dm, Ce, Sc, Ec, Bs	Target sequence (<5 kbp), gene name	Mismatches	[62]
Protospacer Workbench	Any species	Target sequence (>10 kbp), gene name	Mismatches	[63]

Hs: *Homo sapiens*, Rn: *Rattus norvegicus*, Mm: *Mus musculus*, Dr: *Danio rerio*, Dm: *Drosophila melanogaster*, Aa: *Aedes aegypti*, Ec: *Escherichia coli*, Sc: *Saccharomyces cerevisiae*, Bs: *Bacillus subtilis*, Ce: *Caenorhabditis elegans*, Mam: *Macaca mulatta*.

The web tool CRISPRdirect searches not only the entire sgRNA sequence for potential off-target sites, but also the seed sequences, such as the first 8 and 12 nt in addition to PAM. This way, users can choose their preferred sgRNA with the minimal off-target sites in the seed region. Perl based stand-alone software CasOT [64] and sgRNAcas9 [65] also consider the number and position of mismatches, for instance, whether the position of the mismatches are located in the non-seed or seed region for ranking the potential off-target sites. In addition, previous studies have shown that sgRNA sequences with extremely high or low GC content (*i.e*., >80% or <20%) are less effective against their targets [66]. To avoid sgRNAs with skewed GC content, CRISPRdirect provides the GC content of the designed sgRNAs.

Further analysis of potential off-target sites for candidate sgRNAs can be searched using web tools, such as Cas-OFFinder [67] and COSMID [68]. These web tools search for 20 nt target sequences adjacent to PAMs (e.g., “NGG”, “NRG”) from an input sequence and then output the number of off-target sites and sequences.

Off-target sites are normally searched based on mismatches rather than indels, as shown in Table 1; however, Bao *et al.* showed that sgRNA with a few indels may induce cleavage [69]. Therefore, it is recommended to search for potential off-target sites with indel allowances in addition to base-pair mismatches. CRISPRdirect, Cas-OFFinder, and COSMID consider both indels and mismatches for searching off-target sites and show the positions of the mismatches and gaps, which may help to predict the potency of off-target mutagenesis. In addition to CRISPR sgRNA dedicated software, sequence search tools (such as Blast, Blat, GGGenome, and TagScan) or short read mapping software (such as Bowtie and BWA) might be useful to search for potential off-target sites. Although these general sequence aligners do not consider the PAM or the seed when scoring similar sequences to sgRNA, they are easy to use for quickly checking the degree to sequence specificity.

Most of the online tools have a low throughput to design multiple sgRNAs, and available organisms are limited. However, recently developed off-line software, such as CasOT [55], sgRNAcas9 [58], and Protospacer Workbench [63], have greater flexibility to select targets from any organism where a genomic sequence is available. Offline software provide greater flexibility and computational speed, but some, such as CasOT [55] or sgRNAcas9 [58], require programming knowledge and familiarity with a command line interface.

On-target DNA cleavage activity of sgRNA is also critical when it comes to designing sgRNAs. Apart from the GC content of sgRNAs mentioned above, there is a preference for the nucleotide immediately adjacent to PAM to be “G”, and not “C” [61,66,70,71]. The epigenetic state of the target genome was indicated to have an influence on Cas9 accessibility and subsequent cleavage activity [71], however, CpG methylation was reported separately to not significantly alter this activity [6].

**Figure 2:**
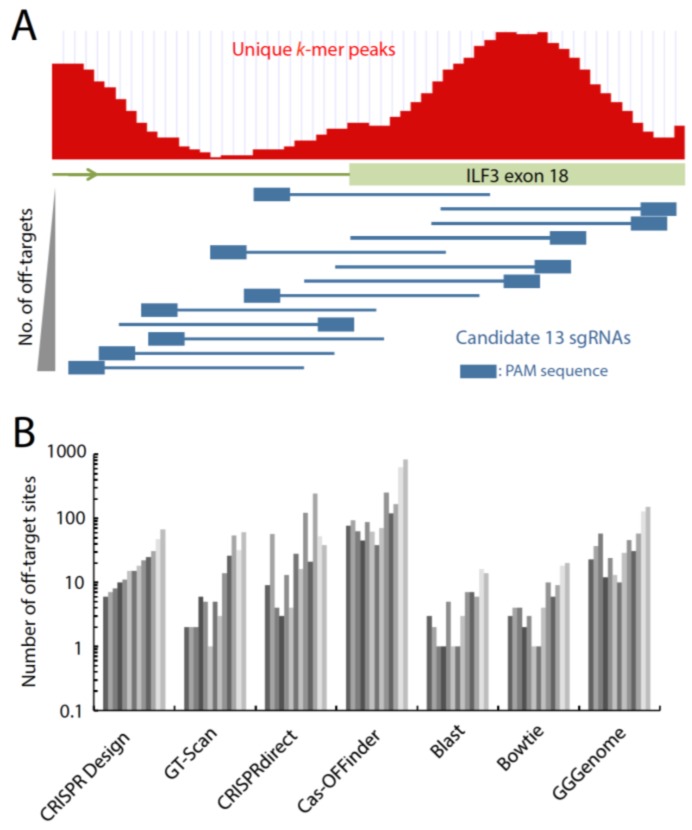
Tendency of identified off-target sites by sgRNA design and sequence search tools. (**A**) To demonstrate the sgRNA design process, we selected the human *ILF3* gene as a target. To identify uniquely targetable region of sgRNAs, genomic region around the splicing acceptor site of exon 18 and peaks of unique *k*-mer sequences are shown as red histogram. Thirteen candidate sgRNA sequences (blue arrow with a box, indicating the position of PAM sequence) were identified within the region and the identified sgRNAs are ordered based on the rank provided by the CRISPR design tool; (**B**) With the same set of 13 sgRNAs as shown in (**A**), we calculated the number of potential off-target sites in human genome (hg19) by using various tools as indicated. For the calculation, the following options are used: GT-Scan, allowing three mismatches; CRISPRdirect, potential targets with 12 nt plus PAM; Cas-OFFinder, allowing three mismatches, two DNA bulge size, and two RNA bulge size; stand alone Blastn+, “blastn-short” task, *e*-value < 1.0; stand alone Bowtie, allowing three mismatches, seed length 16 nt; GGGenome, allowing three mismatches.

In order to simulate the process of selecting sgRNAs, we designed sgRNAs targeting the exon 18 of the human dsRNA binding protein ILF3 gene on chromosome 19 for a hypothetical gene knockout experiment. First, we used iGEATs (*in silico* Genome Editing and Analysis Tools) [72] to visually identify a unique and targetable region across the exon 18 of ILF3 gene, based on the peak height of short *k*-mer sequences that mapped to the human genome (Figure 2A). We identified that the beginning of exon 18 would serve as a good target region because unique *k*-mer peaks are abundant. Then, we used the CRISPR Design tool to extract candidate sgRNA sequences from the query target sequencing (around 60 bp region) around the splicing acceptor site of exon 18 of ILF3. We selected all 13 sgRNA sequences from the CRISPR Design output, and calculated the number of potential off-target sites by GT-Scan, CRISPRdirect, and Cas-OFFinder. We also used general sequence search tools, blastn+ [73], Bowtie [74], and GGGenome to search for the number of potential off-target sites, including insertions or deletions (Figure 2B). Although the computational speed to obtain results was relatively slow, the CRISPR design tool showed not only the number of off-target sites, but also those within gene coding regions. GT-Scan and CRISPRdirect could rapidly calculate the number of the off-target sites, but could not distinguish whether potential off-target sites exist within gene regions. Notably, different sgRNA design tools identify the same 13 sgRNA sequences within the given query sequence, and the ranking of the sgRNAs were relatively conserved among the four sgRNA design tools we tested. The total number of the identified off-targets greatly varied among different tools, presumably due to the different algorithms and/or parameter stringencies (Figure 2B). Furthermore, the trends of the number of potential off-targets were also conserved among sequence search tools, blastn+ (command line mode, “blastn-short” task, *e*-valuce < 1.0), Bowtie (command line mode, 3 bp mismatch allowance, seed length 16 bp), and GGGenome (3 bp mismatch allowance). Since blastn+ and Bowtie provided less potential off-target sites compared with the sgRNA design tools, some potential off-target sites may have been excluded. These results suggest that the full identification of all off-target sites is challenging by using some of general sequencing mapping software. To determine which off-target prediction method is the most reliable, a more comprehensive comparison of the various tools along side-by-side with actual experimental data is needed.

## 5. Modified Nucleases to Reduce off-Target Mutagenesis

Modulating Cas9 activity through the substitution of amino acid residues involved in its enzymatic function could fine tune substrate recognition and reduce nonspecific DNA cleavage. Alternatively, by completely abolishing the enzymatic activity of Cas9 while retaining its ability to target DNA, a higher fidelity endonuclease could be fused to a deactivated Cas9 homing module to increase on-target cleavage.

**Figure 3:**
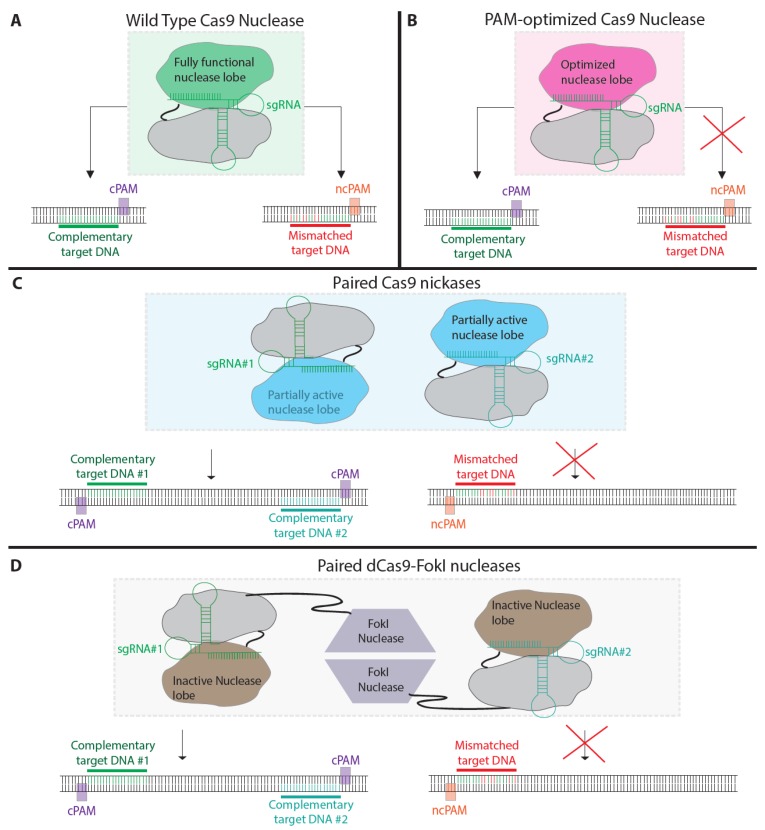
Modified Cas9 strategies to reduce off-target mutagenesis. (**A**) Wild type Sp Cas9 nuclease can bind and cleave either sgRNA-complementary (green) or -mismatched (red) DNA with canonical PAM (cPAM) or noncanonical PAM (ncPAM), respectively; (**B**) PAM-optimized Cas9 nuclease induces cleavage specifically to cPAM complementary DNA; (**C**) A pair of SpCas9 nickases (*i.e*., D10A mutant) must bind to two closely spaced DNA sequences on opposite strands in order to induce single strand nicking on each strand and result in full cleavage of the target DNA. The binding of a Cas9 nickase to a non-specific site will induce nicking but not a double strand break; (**D**) Deactivated Sp dCas9 (*i.e.*, D10A and H840A mutant) fused with a FokI nuclease must bind to two closely spaced DNA sequences in order for dimerization of the FokI nuclease and cleavage to occur. Nonspecific binding at an off-target site will not induce cleavage.

In one strategy, the Cas9 PAM recognition domain can be modified to reduce the number of potential off-target mutagenesis. An *in vitro* assessment of recombinant Cas9 by single molecule imaging revealed that Cas9 complexed with sgRNA scans complementary DNA sequences depending on the presence of possible PAM recognition sites [43]. Sp Cas9 typically recognizes “NGG” but can also use non-canonical PAMs, thereby increasing off target cleavage (Figure 3A). In a recent report, by Joung and colleagues, Sp Cas9 was engineered with a D1135E mutation that increased the specificity of on-target cleavage by reducing non-canonical PAM recognition (Figure 3B) [75]. In addition, the group modified Sp Cas9 to recognize different PAM sequences with different lengths. Such engineering may be able to fine tune Cas9 activity and greatly enhance specificity, which is defined as the ratio of on-site cleavage to off-site cleavage. More recently, Davis *et al.* [76] showed that the suppression of off-target cleavage by insertion of an engineered intein (which is fused with estrogen receptor ligand-binding domain) into Sp Cas9. The addition of 4-hydroxytamoxifen induced protein splicing to remove the engineered intein and activate Cas9. As a result, DNA cleavage specificity improved as high as 25-fold compared with wild-type Cas9.

Two additional mutagenesis approaches have been used to mitigate CRISPR Cas9 off target cleavage activity by attenuating or abolishing its nuclease function. For partial mutagenesis, a known aspartate to alanine mutation (D10A) inactivates the RuvC nuclease domain while leaving the HNH domain intact [1]. The resulting nickase activity cleaves only one strand of the dsDNA, creating a small gap as opposed to a complete blunt dsDNA break, and is subsequently fixed by a less error prone base excision repair (BER) pathway which involves multiple enzymes that recognize and repair the damaged DNA. Single nicking enhances HR events, but is much less effective than DSB mediated HR. Combining two closely spaced sgRNAs, offset by less than 100 bp, that target opposite strands of a DNA target sequence can lead to the efficient induction of DSBs and HR (Figure 3C) [6,77]. The general consensus from several groups has been that the double nickase strategy efficiently cleaves on-target sites as well as the WT Cas9, while lowering off-target mutations [77,78,79]. However, even this method is not completely free of risks as point mutations can result from nickase activity [80].

Fusing a catalytically dead Cas9 protein (D10A and H840A mutations) with a Fok I nuclease, similarly to what has been done with ZFNs [81,82] and TALENs, can add another level of control during genome editing. In doing so, DNA targeting by Cas9-sgRNA is retained and cleavage activity occurs only after FokI dimerization [80,83] To function, two unique sgRNAs targeting DNA sequences, which are spaced approximately 17 base pairs apart, can bring together Cas9-FokI chimeras into close enough proximity of each other to allow for dimerization and subsequent DNA cleavage (Figure 3D). Thus, it is possible to maintain the DNA homing characteristics of CRISPR Cas9 while, at the same time, replacing its off-target behavior with a more specific FokI nuclease [80]. The downside to using FokI is that it is significantly larger than Cas9, meaning that the transduction efficiency of the plasmid DNA could be compromised.

While gene editing experiments to date have focused on Sp Cas9, *Staphylococcus aureus* (Sa) Cas9 has been reported to have high cleavage activity, comparable to that of Sp Cas9. Unlike the “NGG” PAM recognition of Sp Cas9, Sa Cas9 has a longer PAM consisting of “NNGRRN” meaning that the number of off-target candidates could be less [75,84]. However, a larger sample size is needed before any conclusions can be drawn.

## 6. Cas9 Delivery and Expression

To express Cas9 protein and sgRNA, DNA plasmid transfections have been commonly employed in various cell lines for experiments examining on- and off-target cleavage. However, the overexpression of both Cas9 protein and sgRNA by DNA plasmid transfection has been shown to induce off-target mutagenesis when higher amounts of DNA are transfected [6]. Thus, there appears to be an undefined threshold where the levels of Cas9 protein and sgRNA could become “toxic” and lead to a higher occurrence of off-target effects. Intuitively, this makes sense because increasing the number of interaction events increases the probability that Cas9 may bind and cleave an off-target site.

Other methods to temporally express Cas9 in cells include the use of an inducible CRISPR (iCRISPR) Cas9 expression system, and delivery by co-transfecting Cas9 mRNA and sgRNA or Cas9 protein pre-loaded with sgRNA. Stable iCRISPR expressing human pluripotent cells, reported by a study led by Huangfu [85], can transiently express Cas9 under the control of a tetracycline responsive promoter. By transfecting sgRNAs into these cells, efficient knockout of genes could be performed with reduced off-target cleavage. Another approach to limiting Cas9 expression by DNA transfection is to simultaneously target the Cas9 plasmid with an additional sgRNA to prevent prolonged protein expression of the nuclease [86].

Alternatively, because RNA and protein are unstable by nature and have relatively short half-lives, their direct delivery into cells may also reduce the transient time that Cas9 exists, and therefore, limit off-target effects. Indeed, Kim and colleagues [87] showed that the specificity of on-target cleavage *versus* off target cleavage is up to 13 times greater when using Cas9 protein compared with Cas9 plasmid delivery. When the authors examined Cas9 protein levels over a time course of 72 h, protein was undetectable after 24 h, whereas plasmid transfection resulted in peak Cas9 protein levels at 24 h with high levels still detected up to 72 h [87]. Altogether, these results suggest that prolonged Cas9 and sgRNA expression can lead to accumulating mutations.

Recently, chemical modifications at the 3′ end of sgRNAs with 2′-thionocarbanate-protected nucleoside phosphoramidites have been reported to enhance cleavage activity and improve the specificity of some sgRNA [88]. In addition, it is reported that short sgRNA (*i.e*., 17 nt) have higher specificity, as it is less tolerated to mismatches to induce DNA cleavage [89]. Therefore, modifying sgRNA is another option to reduce non-specificity.

## 7. Assessing off-Target Mutagenesis

A robust evaluation of potential off-target sites is necessary to fully assess the extent of non-specific cleavage by Cas9 [90,91]. There are three main categories of assessing methods: (i) search and assess off-target mutagenesis at locations similar to the on-target sgRNA sequence; (ii) assess the genomic regions where DSBs have been induced; and (iii) search the whole genome to check for genomic integrity. In each category, several strategies are available, which vary in sensitivity and coverage of both biased and unbiased sequences. Furthermore, whether to analyze bulk cultured cells or cells isolated by subcloning should be considered to determine the types of mutations that can arise in individual cells types.

For the first category, potential off-target sites are predicted using appropriate software as mentioned above, and then assessed by a mutation detection assay, such as T7EI (T7 endonuclease I) assay or deep sequencing of the PCR products (also known as amplicon seq). For the T7EI assay, PCR primers to amplify the predicted cleavage sites from genomic DNA are designed to be approximately 500 to 1000 bp apart. The amplicons are denatured and slowly re-annealed so that strands containing indels will anneal to uncleaved strands creating mismatches that the T7E1 endonuclease will cleave (similarly in principle, the CEL-I assay or SURVEYOR assay can be used). Any cleavage products run on an electrophoresis are positive indicators of Cas9 activity. Many laboratories use this technique as it is inexpensive and no special equipment is required, but its sensitivity is low and coverage is limited due to specific primers needed for each predicted target sequence (*i.e*., deletions larger than primer binding sites cannot be detected). In addition, homozygous mutations can not be detected, unless non-mutated PCR products are spiked-in. Sanger sequencing of PCR amplicons makes it possible to detect indels with higher sensitivity but suffers from limited coverage of the chosen primer sets.

To look at the off-target binding of Cas9 in an exogenously established system, Church and colleagues fused a catalytically dead Cas9 protein (dCas9) with a transcriptional activator, VP64, to screen a library of sequences that, when bound to dCas9-VP64, activate a fluorescence reporter in HEK293T cells [78]. This study demonstrated the off-target binding affinity of Cas9 protein with sgRNAs containing up to three mismatches. A similar study led by Sander *et al.* [42] used an EGFP disruption assay to test the specificity of CRISPR Cas9 cleavage with sgRNAs harboring up to five mismatches at different positions in the sgRNA. The authors used HEK293T cells stably expressing EGFP, and examined gRNA candidates that diminished EGFP expression.

SELEX (systematic evolution of ligands by exponential enrichment) is an *in vitro* method that investigates the binding of Cas9, or any nuclease, to a library of oligonucleotides to anticipate potential off-target sites under controlled conditions [41,92]. Genomic DNA sequences that match those of the bound oligonucleotides can then be searched.

An interaction approach by chromatin immunoprecipitation sequencing (ChIP-seq) takes advantage of binding by dCas9 to DNA sequences for assessing potential off target sites [41,93,94]. However, it is worth mentioning that a comparison between the off-target identification using CHiP Seq with catalytically dead Cas9 and catalytically active Cas9 did not correlate well, meaning that the binding affinity of Cas9 to target sites does not always lead to cleavage [93].

As the second category of off-target analysis, a DSB event itself can be tagged or captured to look for potential off-target mutagenesis by deep sequencing. One such method is to use integration-defective lentiviral vectors (IDLVs) that are incorporated into DSBs induced by Cas9 [95]. The vector generally contains a selection marker such as puromycin resistance, which can be used to enrich the population of cells with incorporated IDLVs. The technique has been used for ZFNs, TALENs, and CRISPR Cas9 but has a sensitivity of 0.5% and may be underestimating the total number of DSBs.

Similarly to map genome-wide DSBs, BLESS (direct *in situ* breaks labeling, enrichment on streptavidin and next-generation sequencing) captures a snapshot of DSBs in fixed cells by labeling the DSB sites with biotinylated linkers and deep-sequencing after PCR amplification of the captured DNA fragments [96]. The technique has been successfully applied to assessing Sa Cas9 off-target cleavage in 293FT cells, which are derived from HEK293 cells [84].

In a more sensitive assay to capture DSBs in live cells, GUIDE-seq (Genome-wide, unbiased identification of DSBs enabled by sequencing) is a method that tags DSBs by incorporating short phosphorylated double-stranded oligodeoxynucleotides into them [97]. The regions are amplified by PCR and then read by next generation sequencing. This technique is sensitive enough to detect mutagenesis that occurs at a rate of 0.1%. To detect DSB mediated chromosomal translocation, the HTGTS (high-throughput, genome-wide, translocation sequencing) method has been reported [94]. Linear amplification mediated (LAM)-PCR amplifies the region near the nuclease target site from one direction, and searches for translocated chimeric sequencing reads from deep sequencing data. Another unbiased approach called digenome-seq (digested genome) subjects genomic DNA, extracted from untreated or Cas9 transfected cells, to cleavage by recombinant Cas9 and sgRNA *in vitro* [98]. By comparing both sample reads from whole genome sequencing data, a pattern will emerge where uncleaved and cleaved sites can be distinguished. Like GUIDE-seq, the sensitivity of the assay is as high as 0.1%, however, high-coverage WGS data is required to fully achieve the sensitivity. The overall genomic stability in edited cells can be affected by both sgRNA-dependent and -independent events. For example, sgRNA-independent abnormalities can be caused from various forms of stress induced by experimental conditions such as transduction of Cas9-sgRNA complexes.

As the third category of the assay to detect off-target mutagenesis, genomic integrity can be examined by various methods. This category of the assays is much more relevant for clinical applications, as proof of genomic integrity can be a minimal requirement. Genomic abnormalities induced by CRISPR sgRNAs and/or other means can be highly variable in terms of their sizes. For example, chromosomal abnormalities, such as deletions, duplications, inversions, or translocations of larger than 1 Mb, karyotyping is a sensitive and established assay to detect minor cell populations with chromosomal abnormalities (*i.e*., >10%–20% of cells). However, the assay relies on imaging of chromosomes, and resolution is very low.

The cleavage of DNA at two separate sites in the genome may lead to a loss of large DNA segments, as long as 1 Mbp [99]. For this kind of large deletion, the DNA copy number variance (CNV) should be examined in both the parental and CRISPR Cas9 treated cells [17]. Since CNV analysis uses array technology to detect genomic copy number in a cell population, resolution is higher than karyotyping (~1 kb), but sensitivity is low so that at least >30%~40% of cell population must have a similar CNV to be detected reproducibly.

Investigation of mutations at single base-pair resolution requires the use of deep sequencing. Exome sequencing is a method to capture protein coding regions of the genome and analysis by deep sequencing. Coverage of regions is limited, however, higher coverage can be achieved than whole genome sequencing at a lower cost. In addition, a consequence of the detected mutation can be inferred more easily than other methods, as effect in amino acid coding can be evaluated. Several groups have been using exome sequencing to identify potential off-target mutagenesis in genome edited cells [17].

For a more comprehensive survey of off-target mutagenesis, whole genome sequencing (WGS) has been performed for cell lines [100,101,102] and mice [103] edited by TALENs and CRISPR Cas9. So far, no severe off-target mutagenesis attributed to the nucleases has been found. WGS is a powerful tool to detect genome-wide single nucleotide variations and small indels, but to detect larger deletions (*i.e*., CNV), specific analysis pipelines or algorithms need to be employed. This may have led to the exclusion of real target sites that were confused with artifacts during sequencing analysis or a lack of sensitivity to detect low frequency mutants that occur in less than 10% of clones. In sum, the combination of several off-target assays should be considered to fully assess the risk of off-target mutagenesis.

## 8. Off-Target Analysis Based on Genome Editing Objectives

How stringently off-target cleavage should be considered when using genome editing tools will depend on the research aim of the investigating scientist. For basic research (*i.e*., gene knockout, reporter knock-in) using cell lines, off-target mutagenesis should be minimized by careful design of sgRNAs, but it is not an absolute requirement so long as the scientific conclusions are supported by multiple sgRNAs or clones, as it is unlikely that the same off-target mutagenesis will occur in multiple clones. For the clinical setting, using *ex vivo* gene therapy approaches, sgRNA must be carefully designed to have minimum off-target cleavage risks. However, even with an optimized experimental design, initial clinical trials should rigorously examine the genomic integrity of the edited cells. Detection of DSB sites by massive parallel sequencers is a powerful approach, however, the genomic integrity cannot be guaranteed whenever there is a DSB involved. Classical karyotyping, CNV analysis, and exome sequencing, or whole genome sequencing might be required to show that the genomic DNA in the genome edited cells is intact. For an *in vivo* gene therapy approach, a complete and rigorous design of sgRNA is required. However, verifying the genomic integrity of the treated cells *in vivo* is much more challenging.

## 9. Target Cells Matter

There have been conflicting reports in the literature about the detection of off-target cleavage by engineered nucleases, in particular TALENS and CRISPR Cas9, in different cell types (Table 2). Interestingly, in the majority of studies that detected off-target mutagenesis, nearly all of the experiments were performed in cancer cell lines such as HEK293, U2OS, and K562. On the other hand, in reports where off-target mutations were undetected, pluripotent stem cells (ES and iPS cells) were used. It is important to note that while these studies cannot be compared side by side because the experimental parameters and methods used to detect the off-target mutations were not identical, the different results may indicate a higher propensity of observed off-target mutations to occur in cancer cell lines where the DNA repair machinery has been compromised. In fact, Joung and colleagues identified DSB “hot spots”, or regions that are likely to have DSBs, independent of CRISPR Cas9 in HEK293 and U20S cells [97]. On the contrary, several lines of studies identified pluripotent stem cell specific “genomic guardians”, such as Zscan4 [104], to maintain genomic stability. Future studies will elucidate whether there is a correlation between off-target mutagenesis and the cell type being edited.

**Table 2:** Summary of studies detecting off-target mutagenesis using different cell lines.

Cell Types	Target Gene	Programmable Nuclease Used	Detection Assay of off-Target Mutagenesis	off-Target Mutagenesis Detected	Ref.
U2OS, HEK293 and K562 cells	*VEGFA*, *EMX1*, *RNF2*, *FANCF*	CRISPR	EGFP reporter, T7EI	Yes	[42]
293T	*EMX1*	TALENs, CRISPR	amplicon seq, Surveyor assay	Yes	[6]
293T	*HBB*, *CCR5*	CRISPR	T7EI, Sanger sequencing	Yes	[105]
293T	*HBB*, *CCR5*	CRISPR	T7EI, Sanger sequencing	Yes	[69]
293 and U2OS cells	*VEFGA*, *EMX1*, *FANCF*, *RNF2*	CRISPR	GUIDE-seq, amplicon seq (AMP-based seq)	Yes	[97]
HAP1 cells, K562 cells,	*HBB*, *VEFGA*	CRISPR	Digenome-seq	Yes	[98]
293T cells, A549 cells	*RAG1*, *C-MYC*, *ATM*	TALENs, CRISPR	HTGTS method	Yes	[94]
iPS cells	*A1AT*	ZFNs	CGH, SNP array, exome seq	No	[106]
myoblasts	*DMD*	TALENs	exome seq	No	[107]
iPS cells	*PPP1R12C*	TALENs, CRISPR	whole genome seq	No	[100]
ES cells, iPS cells	*SORT1*, *LINC00116*	TALENs, CRISPR	whole genome seq	No	[102]
iPS cells	*HBB*	TALENs, HDAdV mediated HR	whole genome seq	No	[101]
293FT cells, iPS cells	*PPP1R12C*, *AKT2*, *CDK19*, *ATP6AP2*, *SLC35A2*	CRISPR	amplicon seq	No	[108]
iPS cells	*DMD*	TALENs and CRISPR	T7EI, amplicon seq, Karyotyping, CNV analysis, exome seq	No	[17]
iPS cells	*PLN*	TALEN	exome seq	No	[109]
iPS cells	*F8*	CRISPR	amplicon seq	No	[21]

## 10. Conclusions

In conclusion, there are several considerations to take into account in order to minimize off-target mutagenesis when using the CRISPR Cas9 system. The sgRNA design is critical to avoid similar sequences, and numerous online tools are available to select highly specific sgRNA complementary sequences. A modified Cas9 nuclease can also be selected for targeting purposes, such as the paired D10A Cas9 approach over the wild type nuclease. In addition, further enhancements to the PAM recognition motif may help to increase canonical PAM specificity while reducing the non-canonical PAM promiscuity. Finally, the detection method used will be important to discover low frequency mutagenesis at off-target sites. However, in considering these options, the need for specificity will depend on the research aims. Basic researchers using cancer cell lines will have less interest in conducting whole genome sequencing in order to identify off-target mutagenesis, whereas a researcher aiming to correct a genetic disease for clinical applications may be required to minimize off-target risks, such as tumorigenesis, in treated patients.
